# Current Strategies for Treating NSCLC: From Biological Mechanisms to Clinical Treatment

**DOI:** 10.3390/cancers12061587

**Published:** 2020-06-15

**Authors:** Junnan Li, Hang Fai Kwok

**Affiliations:** 1 Cancer Centre, Faculty of Health Sciences, University of Macau, Avenida de Universidade, Taipa, Macau; yb97607@um.edu.mo; 2 Institute of Translational Medicine, Faculty of Health Sciences, University of Macau, Avenida de Universidade, Taipa, Macau

**Keywords:** lung cancer, drug resistance mechanism, EGFR mutation, combination targeted therapy

## Abstract

The identification of specific epidermal growth factor receptor (EGFR)-activating mutations heralded a breakthrough in non-small-cell lung cancer (NSCLC) treatments, with the subsequent development of EGFR-tyrosine kinase inhibitor (TKIs) becoming the first-line therapy for patients harboring EGFR mutations. However, acquired resistance to EGFR-TKIs inevitably occurs in patients following initial TKI treatment, leading to disease progression. Various mechanisms are behind the acquired resistance, and mainly include (1) target gene modification, (2) alternative parallel pathway activation, (3) downstream pathway activation, and (4) histological/phenotypic transformation. Approaches to combat the acquired resistance have been investigated according to these mechanisms. Newer generations of TKIs have been developed to target the secondary/tertiary EGFR mutations in patients with acquired resistance. In addition, combination therapies have been developed as another promising strategy to overcome acquired resistance through the activation of other signaling pathways. Thus, in this review, we summarize the mechanisms for acquired resistance and focus on the potential corresponding therapeutic strategies for acquired resistance.

## 1. Background

Lung cancer remains one of the most malignant cancers in the world, with about 1.8 million reported lung-cancer-related deaths in 2018, accounting for nearly one-fifth of all cancer-related deaths that year [1]. About 85% of those cases are diagnosed as non-small-cell lung cancer (NSCLC), and up to 84% of those NSCLC patients suffer from advanced stage cancer and present with metastasis [2]. Among these NSCLC patients, an epidermal growth factor receptor (EGFR)-activating mutation is present in up to 50% Asians and 15% Caucasians, representing the underlying cause. A decade ago, platinum-based chemotherapy in combination with another chemotherapeutic agent (pemetrexed, gemcitabine, or vinorelbine) was the conventional therapy, but it did not produce satisfactory effects [3].

Receptor tyrosine kinases (RTKs) are a type of transmembrane receptor characterized by an extracellular ligand-binding region, a single hydrophobic transmembrane domain, and an intracellular domain within the tyrosine kinase region. Members of the transmembrane EGF receptor family are one type of RTK. The EGF receptor family includes ErbB1 (or EGFR), ErbB2 (or HER2), ErbB3 (or HER3), and ErbB4 (or HER4) [4]. Upon binding to their respective ligand, the conformation of these receptors changes, transforming them into an active form that is associated with homo- or heterodimerization. Then, the key tyrosine residues autophosphorylate, activating downstream signaling pathways and promoting cell migration, proliferation, and other processes [4]. The identification of a specific EGFR-activating mutation led to a breakthrough in the development of NSCLC treatments. The short in-frame deletions in exon 19 and point mutation in exon 21 (Leu858Arg) are the most common activating mutations found in EGFR [5]. Regarding treatment, the development of EGFR- tyrosine kinase inhibitor (TKIs) resulted in some success, though it proved to be short-lived, as acquired resistance to these TKIs inevitably develops as a result of secondary or tertiary EGFR mutations.

In addition to EGFR, other types of receptor tyrosine kinases, such as mesenchymal–epithelial transition factor (MET), hepatocyte growth receptors (HGFs), insulin-like growth factors receptors (IGFRs), fibroblast growth factor receptors (FGFRs), and vascular endothelial growth factor receptors (VEGFRs), can contribute to the activation of a bypassing or downstream signaling pathway, also leading to acquired resistance [6]. Better strategies are urgently needed to combat acquired resistance, and this requires the mechanisms of drug resistance to be uncovered and characterized in detail. In this review, we summarize the knowledge regarding mechanisms of drug resistance and outline the latest therapeutic strategies used to overcome this problem.

## 2. Primary Resistance

Even though most NSCLC patients with EGFR mutations achieve an objective response (OR) to TKIs, 20–30% of patients still show disappointing outcomes after TKI treatment due to intrinsic resistance [7]. One of the mechanisms behind intrinsic resistance is the differential sensitivities of the various TKIs to different EGFR mutations. The in-frame deletion in exon 19 and L858R point mutation in exon 21 are the most common somatic mutations in NSCLC patients, accounting for about 80% of the total. More than 20 unique deletions in exon 19 have been detected in patients who show longer median survival times during EGFR-TKI treatment than those harboring the L858R point mutation in exon 21. Other nonclassical, sensitizing mutations (mainly exon 20 insertion) can induce intrinsic resistance. Intrinsic resistance may also arise due to intrinsic secondary genetic alternations. A resistant clone (e.g., T790M) may pre-exist in cell clones and lead to the development of drug resistance during treatment [8]. Other studies reported that in almost 1% of lung cancer patients, 2–3 concurrent driver mutations were detected before treatment. Some coexistence of molecular and genetic alternations, such as the loss of K-Ras/PTEN expression, have been observed in intrinsic resistance [9]. These pre-existing molecular and genetic alternations can stimulate the Ras-Raf-MEK-ERK and PI3K/AKT downstream pathways, respectively, and drive cancer cell survival.

## 3. Acquired Resistance and Corresponding Strategies

**Figure 1:**
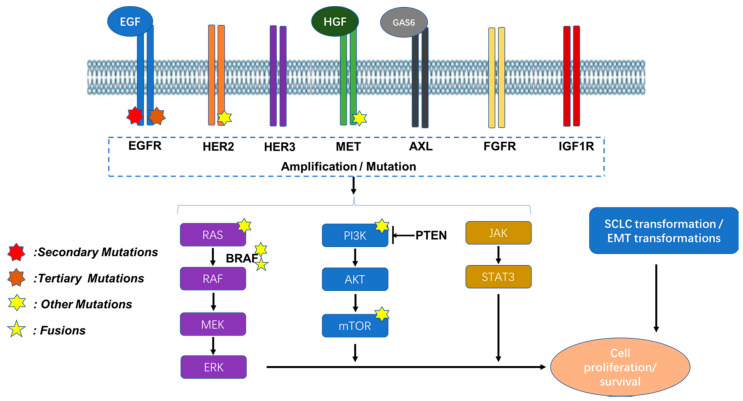
The molecular mechanisms for acquired resistance. The mechanisms include target gene modification, alternative parallel pathway activation, downstream pathway activation, and histological/phenotypic transformation. Both the amplification and mutations of receptor tyrosine kinases (RTKs) can induce downstream survival signaling pathways. Moreover, the direct overexpression and/or mutations of components of downstream pathways can also contribute to acquired resistance by assisting tumor cell survival. These mechanisms provide potential targets for combination strategies for treatment in cases of acquired resistance.

Patients who respond to EGFR-TKIs initially experience good clinical therapeutic effects, though, inevitably, after 11–13 months of treatment, some new resistance mechanisms develop as the tumor cells adapt to the treatment, which is termed “acquired resistance”. Four main types of mechanisms can contribute to acquired resistance: (1) target gene modification, (2) alternative parallel pathway activation, (3) downstream pathway activation, and (4) histological/phenotypic transformation. The molecular mechanisms are depicted in Figure 1 and are each discussed below.

### 3.1. Target Gene Modification

#### 3.1.1. Progression of Mutation and Newer Generations TKIs

With the identification of specific EGFR mutations, several generations of EFGR-TKIs have been developed. To better understand these TKIs, we summarize their characteristics in Table 1.

##### First-Generation EFGR-TKIs

The first-generation EFGR-TKIs include erlotinib, gefitinib, and icotinib. These TKIs can reversibly compete with ATP for binding by forming a hydrogen bond with Met793 in the ATP-binding pocket of EGFR. Numerous phase III trials have demonstrated the robust efficacy of the first generation of EFGR-TKIs, including the erlotinib trials, OPTIMAL and EURTAC, as well as the gefitinib trials, First-SIGNAL (subgroup analysis), WJTOG3405, and NEJ002 [10,11,12,13]. These trials demonstrated that the first-generation EGFR-TKIs significantly improve PFS compared with platinum-based chemotherapy, with a median PFS of 9.2–11.1 months with EGFR-TKIs vs. 4.6–6.9 months with platinum doublets. In the WJOG5108L clinical trial, gefitinib and erlotinib showed similar effects on PFS [14]. A comparable trend was also observed in the ICOGEN study, with no significant difference between PFS and OS in patients treated with gefitinib and icotinib [15]. The objective response to first-generation TKIs is up to 70% in patients with EGFR mutations, but acquired resistance is inevitable after 9–12 months of treatment. The most common modification after the treatment of first-generation TKIs is the secondary EGFR T790M mutation, which accounts for 50–60% of patients with acquired resistance [16]. The mutation occurs at position 790 in exon 20, in which threonine is substituted with methionine. Threonine 790 is the gatekeeper for EGFR because it is located at the entrance to the hydrophobic ATP-binding pocket; this mutation promotes cancer cell proliferation via increasing the catalytic activity of EGFR [17]. The substitution hinders the drug binding via increase in the spatial structure in addition to enhancing the EGFR’s affinity for ATP over EGFR-TKIs, resulting in the low potency of TKIs. Other rare mutations in EGFR have also been reported, such as G719X, S768I, or L861Q, but their underlying mechanisms remain unclear [18,19].

##### Second-Generation TKIs

Afatinib and dacomitinib are second-generation EFGR-TKIs. They have the same quinazoline backbone as the first-generation TKIs, but their side chain can irreversibly bind the cysteine 797 residue, resulting in the irreversible inhibition of EGFR tyrosine kinase. Afatinib was studied as a first-line treatment and compared with chemotherapy for NSCLC patients with EGFR-activating mutations in two large phase III trials. In both trials, afatinib showed a higher RR (56 vs. 23; 66.9 vs. 23.0) and PFS (11.1 vs. 6.9; 11 vs. 5.6) compared to chemotherapy [20,21]. In the LUX-Lung7 clinical trial, afatinib and gefitinib were used as a first-line treatment for NSCLC patients carrying an EGFR mutation. The results showed that afatinib prolonged PFS (11.0 months) more than gefitinib (10.9 months) and reduced the risk of lung cancer progression and treatment failure by 27%. Moreover, afatinib significantly improved the time-to-treatment failure compared with gefitinib [22]. However, both are non-selective; thus, in addition to binding EGFR, they also inhibit pan-HER, resulting in a low maximum tolerated dose and numerous adverse reactions in patients [23,24,25]. The emergence of acquired resistance, as is the case with first-generation TKIs, is also the primary obstacle to the clinical application of afatinib, and a T790M mutation is the main mechanism resulting in acquired resistance to afatinib.

##### Third-Generation TKIs

Various third-generation EFGR-TKIs, such as osimertinib, rociletinib, lazertinib, abivertinib, nazartinib, and olmutinib, have been developed, demonstrating irreversible, mutation-selective, and good activity in cases of primary activating mutations and the resistance mutation T790M [26]. At present, osimertinib is approved for clinical use by the Food and Drug Administration (FDA). The efficacy of osimertinib was demonstrated in the AURA3 trial, which was conducted in NSCLC patients with advanced progression following first-line EGFR-TKI therapy due to the becoming positive for the T790M mutation in EGFR [27]. When comparing the osimertinib treatment group to the chemotherapy (pemetrexed plus platinum-containing drugs) group, the ORR for osimertinib was significantly higher than that for chemotherapy (71% vs. 31%), and the mPFS was also longer in the osimertinib group (10.1 vs. 4.4 months) [27]. The efficacies of osimertinib and first-generation TKIs were compared in the FLAURA clinical trial, which included 556 treatment-naïve patients with advanced or metastatic cancer. The results showed that osimertinib prolonged mPFS remarkably more than erlotinib/gefitinib [28]. First- and second-generation EFGR-TKIs cannot cross the blood–brain barrier (BBB) effectively, whereas osimertinib demonstrated a greater penetration of the BBB than other generations of TKIs in preclinical studies. At the data cut-off point (15 April 2016), the ORRs were 70% and 31% for NSCLC patients with brain metastasis who received osimertinib and platinum/pemetrexed, respectively. The medium duration of the CNS PFS was also longer in the osimertinib group than in the chemotherapy group (11.7 vs. 5.6 months) [29]. Based on all the data, osimertinib can be administered at both initial diagnosis and during advanced disease progression.

Clinical trials were also conducted on other third-generation TKIs. For example, lazertinib was tested in a clinical trial NCT03046992; the primary endpoints were safety and tolerability, and the phase II extension study is ongoing [30]. Abivertinib is a mutation-selective third-generation TKI, and its well-tolerated safety profile and anticancer activity were demonstrated in phase I/II studies (NCT02330367) [31]. Although those TKIs achieved temporary success, a tertiary mutation, C797S, has become a further challenge hampering clinical treatment. This mutation is located at the binding site of EGFR, in which the cysteine at position 797 is substituted with serine [32]. Since the third-generation TKIs also target this site and irreversibly bind to cysteine 797 residue, this mutation allows tumors to gain resistance to all third-generation TKIs. About 40% of patients with T790M also harbor the C797S mutation after osimertinib treatment.

##### Fourth-Generation TKIs

The fourth-generation TKIs, such as EAI045, were produced to overcome the C797S mutation [33]. EAI045 is an allosteric inhibitor based on the thiazole amide EAI001 that can specifically target the C797S mutation. The crystal structure shows that EAI045 binds to an allosteric site created by the displacement of the regulatory C-helix in an inactive conformation of kinase and then inhibits EGFR activation. However, the use of EAI045 alone is ineffective as the asymmetric dimerization of EGFR affects the active state and results in decreased potency. The combination of EAI045 with cetuximab, an antibody that blocks EGFR dimerization, renders the kinase sensitive to EAI045 and was effective in a mice model of lung cancer driven by EGFR(L858R/T790M)- and EGFR(L858R/T790M/C797S) mutations [34]. However, the clinical efficacy of this TKI remains unknown.

#### 3.1.2. Sequential Therapy

Various reports have demonstrated that different generations of TKIs have different efficacies and tolerability. Since gefitinib, erlotinib, and afatinib show better a RR and PFS than traditional chemotherapy in patients with the EGFR mutation, they were approved as first-line treatments [35,36,37]. Osimertinib, which is always used as a second-line treatment option, has also been strikingly efficacious in TKI treatment-naïve patients [38]. In the phase I AURA trial, 60 patients with the EGFR mutation (only 23% with the T790M mutation) received 80 or 160 mg/day osimertinib treatment. The ORR was 67% and 87%, and PFS was 22.1 and 19.3 months, respectively [39]. These results are more in line with the efficacy of erlotinib or afatinib in the first line, indicating that osimertinib can serve in the first-line treatment for NSCLC.

After first-line afatinib therapy, patients are always administered subsequent therapy such as osimertinib. The feasibility of sequential therapy was proven by a post-host analysis of clinical studies. The outcome for the patients receiving osimertinib after the discontinuation of afatinib is encouraging. After a median follow-up of 4.7 years, the median OS had not been reached, and the four-year survival rate was approximately 90% [40]. However, no specific treatment exists for patients who suffer from osimertinib resistance, and most of them will receive chemotherapy. In a preclinical study, third-generation resistance was established in T790M-positive cell lines. This resistant cell line acquired an additional C797S mutation after persistent treatment with third-generation TKIs. The cells will be resistant to third-generation TKIs but will still be sensitive to first-generation TKIs if C797S developed in the trans of the T790M allele. However, if the C797S and T790M mutations are in cis, the cells will be resistant to all of the existing EGFR-TKIs [34,41]. This provides a new sequential regimen of using osimertinib in the first line, followed by first-generation TKIs to overcome the trans C797S mutation. Further studies should be conducted to demonstrate the efficacy of this approach.

### 3.2. Parallel Signaling Pathway Activation

Not all cells become TKI resistant via the EGFR mutation. Cells appear to acquire resistance through different patterns. For example, 40% of patients harbor the C797S mutation after osimertinib treatment, whereas less than 3% of patients present with the C797S mutation after rociletinib treatment. Intra-patient heterogeneity is always found in rociletinib-resistant patients, which involves MET, PIK3CA, ERRB2, KRAS, and so on [42]. This suggests that many other mechanisms are responsible for acquired resistance and should be identified. Hence, we summarize these mechanisms and list the potential targets and drugs in Table 2.

#### 3.2.1. HER2

HER2 amplification is the common parallel bypass mechanism for acquired resistance, especially for the first and second generations of TKIs. About 10–15% of patients with acquired resistance present with HER2 amplification after TKI treatment [43]. To demonstrate the correlation between HER2 amplification and acquired resistance, HER2 was amplified by introducing HER2 cDNA into TKI-sensitive cell lines, which resulted in resistance to erlotinib in those cell lines. HER2 mutations are also regarded as a potential mechanism for TKI resistance [44]. For example, a novel mechanism, based on a HER2D16 splice variant, was found to contribute to acquired resistance. However, HER2D16 has only previously been reported in breast cancer, where it is known to endow oncogenic capability through Scr kinase signaling. Hus et al. reported a patient who was initially sensitive to osimertinib but eventually experienced progression due to resistance, and the cause was determined to be exon 16-skipping HER2, as identified in plasma [45]. This was further demonstrated in vitro by HER2D16-expressing H1975 cells gaining resistance to osimertinib. In a preclinical study, Hus et al. found that HER2D16 can form heterodimers as well as cooperate with EGFR to form a disulfide-bonding homodimer that then activates downstream signaling to confer acquired resistance to osimertinib. Resistance driven by HER2D16 occurs in an Scr-independent manner [45]. Other mutations occurring in HER2 exon 20 were also observed in patients. HER2 exon 20 mutations include point mutations, such as G776C and L755S, or insertions which lead to downstream activation and provide us with a new target for developing treatment in situations of resistance [46,47].

#### 3.2.2. HER2-Targeted Combination Treatment

Since preclinical and clinical studies in HER2 aberrations are rare, there is no standard of care for patients with HER2 aberrations. Liu et al. focused on the efficacy of osimertinib in mice with HER2 alterations and demonstrated that osimertinib has a robust antitumor efficacy in a HER2 overexpression lung cancer model [48]. Trastuzumab emtansine (T-DM1), a HER2-targeted antibody–drug conjugate, is a novel therapeutic format that conjugates a human antibody with a highly cytotoxic molecule through chemical linkers. The efficacy and safety were investigated in a phase II study in patients with advanced overexpressing HER2. The highest response rates to T-DM1 were found in advanced NSCLC patients with HER2 overexpression (IHC 3+) [49]. In another phase II clinical trial, 6 of 11 NSCLC patients with HER2 exon 20 mutation responded to T-DM1 [50]. Recently, a complex resistance case was reported of a patient harboring T790M, a HER2 mutation, and HER2 amplification after first-line TKI treatment. After a combination treatment with osimertinib and trastuzumab, the patient achieved a clinically meaningful and clear molecular response [51]. This represents a new molecular targeted combination therapy for NSCLC patients with HER2 aberrations. Other HER target inhibitors, such as neratinib and PCC0208027, are also under-investigated, and further studies may provide more opportunities for treatment [52,53].

#### 3.2.3. MET

The abnormal activation of MET is regarded as another leading mechanism for acquired resistance. It mainly includes two mechanisms leading to acquired resistance: MET exon 14-skipping mutation (METex14 mutation) and *MET* amplification. When the METex14 mutation occurs, the ubiquitin ligase-binding sites are missing, resulting in the decreased ubiquitination of receptors and the sustained activation of MET, which contributes to the survival of tumor cells and acquired resistance [54]. This alternation accounts for 4% of lung adenocarcinoma. *MET* amplification has also been frequently observed as resulting in resistance to EGFR-TKI treatment, occurring in about 5–20% of TKI-resistant patients. In the HCC827 cell line, MET can drive the dimerization and phosphorylation of HER3 and then, in turn, activate downstream signaling to compensate for the effect of gefitinib [55]. It can also interact with HER2 and ALK, resulting in the activation of downstream signal cascades. An experimental study suggested that the *MET* gene amplification can promote drug resistance via a MAPK/ERK activation after third-generation TKIs which is independent of EGFR. MET is a tyrosine kinases receptor that can be activated by the ligand hepatocyte growth factor (HGF) and participate in the activation of the PI3K/AKT and RAS/MAPK pathways. The overexpression of HGF and abnormality of the HGF/MET axis can also lead to TKI resistance [56]. This mechanism is reported to be specific because it motivates PI3K/AKT in a HER3-independent manner. Clinically, about 3% of patients harbor MET amplification before treatment [57]. HGF can upregulate pre-existing MET-amplified clones after persistent drug-selective stimulation. These MET-amplified tumor cells tend to be dominant clones and lead to TKI resistance [58]. Thus, MET signaling activation by amplification or by the HGF ligand are unique bypass mechanisms for TKI resistance, which suggests that finding HGF/MET antagonists may be an efficient approach for resistance therapy. EGFR amplification is always accompanied by EGFR T790M, which raises the question as to whether tumor cells can amplify EGFR to promote drug resistance or to circumvent the deleterious effect of T790M [23].

#### 3.2.4. MET-Targeted Combination Treatment

At present, various MET inhibitors have been produced and are under preclinical development. Tivantinib is type of non-ATP-competitive MET inhibitor that was investigated in vitro. Combined with afatinib, it can induce cell apoptosis and significantly inhibit cancer growth [59]. A recently developed novel MET antibody drug conjugate, SHR-A1403, was reported to effectively overcome osimertinib resistance in cancer cells overexpressing MET [60]. Other MET inhibitors that are used in the clinic, such as capmatinib, crizotinib, and savolitinib, were evaluated in combination treatments. Savolitinib is a type Ib potent selective MET inhibitor. In a multicenter, open-label, phase Ib TATTON study (NCT02143466), the combination of osimertinib plus savolitinib was used in the treatment of NSCLC patients harboring an EGFR mutation and MET amplification following advanced TKI treatment, which resulted in an acceptable risk–benefit profile and encouraging antitumor activity outcomes. In the 46 patients progressing to first- or second-generation TKIs, the ORR was 52%, with grade ≥3 adverse events in 43% of cases at the data cut-off (February 2018). In the TATTON phase Ib trial, the ORR was 28%, with grade ≥3 adverse events in 23% of cases in the 48 patients progressing to third-generation TKIs [61,62]. Another application of the MET combination strategy is capmatinib plus gefitinib, which was investigated in patients experiencing disease progression after TKI treatment. Increased activity was observed especially in patients with a high MET expression, resulting in a phase II ORR of 47% [63]. The GEOMETRY duo-1 trial (NCT02468661), a phase Ib/II study focusing on the efficacy of capmatinib plus erlotinib vs. platinum plus pemetrexed in EGFR-TKI-treated NSCLC patients with the MET amplified is still ongoing. It was reported to be effective in patients with the METex14 mutation. The GEOMETRY mono-1 trial, a multicohort phase II study, focused on the efficacy and safety of capmatinib in patients with the METex14 mutation. The results showed a clinically meaningful ORR of 39.1% and 71.4% in patients who had received one to two prior lines of treatment and without previous treatment, respectively [64]. The combination of erlotinib and crizotinib was used in the clinic. A NSCLC patient with an EGFR L858R mutation was resistant to erlotinib after first-line treatment and exhibited MET amplification. Then, a full dose of osimertinib and crizotinib was administered to the patient and the patient demonstrated a sustained partial response with excellent tolerance [65]. Thus, the combination of MET inhibitor with EGFR-TKIs is a promising treatment for patients with MET dysregulation, and these studies provide varied evidence for us to define more effective therapeutic strategies for patients.

#### 3.2.5. AXL

AXL is also a member of RTKs that regulate cell survival, proliferation, metastasis, and other processes. Aberrations of AXL can confer acquired resistance to TKIs via the activation of relevant downstream signaling pathways. The upregulation of AXL was detected in one-fifth of patients with acquired resistance to EFGR-TKIs [66]. Both in vitro and in vivo studies have demonstrated that the aberrant activation of AXL can contribute to erlotinib resistance. In a tumor xenograft mice model of acquired resistance to erlotinib, microarray expression profiling results showed that AXL is highly expressed in resistant tumors. The established erlotinib-resistant HCC827 cell lines also showed high levels of AXL and its ligand GAS6 [67]. A recent study showed that AXL can also confer intrinsic resistance to osimertinib because osimertinib can trigger AXL activation by shutting off a negative feedback loop to SPRY4 [68]. This highlights the role of AXL in intrinsic resistance and provides a target for preventing the emergence of osimertinib-tolerant cells.

#### 3.2.6. AXL-Targeted Combination Treatment

At present, no clinical study has combined AXL inhibitors with EGFR-TKIs; the AXL inhibitors are still under preclinical study. Yuanhuadine (YD) causes the degradation of AXL, resulting in a potent antitumor effect in NSCLC. After treatment with YD, the expression of AXL was effectively downregulated in overexpressed gefitinib-resistant H1299 cell lines. The synergism of YD plus gefitinib showed a strong growth-inhibitory activity in gefitinib-resistant H1299 cell lines [69]. The synergistic effect was also demonstrated in xenograft tumors and patient-derived xenograft models [70]. In the gefitinib- and osimertinib-resistant xenograft mouse model or gefitinib-resistant mice engrafted with patient tumor, YD plus TKI treatment significantly inhibited tumor growth compared with treatment based on YD or TKI alone [70]. Another novel selective inhibitor of AXL, DS-1205b, was reported. The combination of DS-1205b plus erlotinib/osimertinib can effectively inhibit the signaling downstream of EGFR and significantly delay the onset of tumor resistance when compared to monotherapy [71]. The effects of other AXL inhibitors (MGCD265, MGCD516, and R428) were also tested alone or combination with erlotinib in EGFR-TKI-resistant cell lines. The combination strategy can more potently inhibit the growth of resistant cells than single-agent treatment, resulting in G2–M cell cycle arrest and apoptosis [72]. An AXL-specific antibody–drug conjugate was developed, enapotamab vedotin, and shows potent antitumor activity in different NSCLC subtypes in a mouse clinical trial of human NSCLC. Of the 61 patient-derived xenograft models, 17 showed tumor regression or stasis after treatment with enapotamab vedotin; this efficacy was most significant in AXL-expressing NSCLC xenograft models, as was validated in 9 of 10 cases [73]. All of this information provides a novel strategy for the treatment of subsets of patients based on specific biomarkers, and more studies should be conducted in the future to develop these strategies.

#### 3.2.7. Other Bypass Signaling Pathways

Other parallel bypass mechanisms have been reported at low frequency, but they are still worthy of mention. HER3 activation plays a role in the progression of TKI resistance, since it is involved in a crosstalk with MET and other members of the ERBB family. The amplification of FGFR1 was observed in an osimertinib-resistant patient, and the increase expression in *FGF2* mRNA suggests the existence of autocrine loop-mediated resistance to TKIs [74]. Although FGFR inhibitors such as SU5402 and PD166866 exhibit synergistic anticancer activity when combined with erlotinib and lapatinib, respectively, the clinical effects need to be further confirmed [75].

IGF1R plays a role in promoting the growth, survival, and oncogenic transformation of tumor cells via the activation of the RAS/RAF/MAPK and PI3K/AKT signaling pathways. The inhibition of IGF1R was reported to prevent the occurrence of acquired resistance after afatinib treatment [76]. Researchers found that IGF1R can induce TKI resistance by triggering the process of epithelial–mesenchymal transition (EMT) [77]. Although many preclinical studies were conducted to in an attempt to demonstrate the correlation between IGF1R and TKI resistance, research in clinical patients has not yet produced sufficient evidence. Further studies are required for a conclusion.

#### 3.2.8. Other Targeted Combination Treatments

Since angiogenesis plays a fundamental role in tumor progression, many concerns have been raised in VEGF-targeted combination therapy. Preclinical and clinical studies have demonstrated the potential of the combination of VEGF inhibitors plus EGFR-TKIs. Bevacizumab is a recombinant and humanized monoclonal antibody that acts directly against VEGF. The combination treatment with erlotinib was shown to enhance the effect of TKIs in both primary and acquired resistance xenograft models [78]. This strategy was proven to be effective in clinical settings, resulting in prolonging PFS and enhancing ORR in the second-line treatment [79]. The combination therapy of bevacizumab and erlotinib was also proven to have potential efficacy in an NSCLC patient who showed intrinsic resistance to initial TKI therapy. This patient was a 60-year-old male smoker and presented with intrinsic resistance to gefitinib after 28 days of treatment with amplification of MET. After 21 days of combination treatment, his symptoms improved significantly, with shrinking of the tumor [80]. 

Another notable VEGF-targeted combination treatment is erlotinib plus thalidomide. After treatment with erlotinib plus thalidomide in 52 NSCLC patients with resistance to erlotinib, the ORR and DCR were 7.7% and 38.5%, respectively. Further preclinical/clinical studies are required to provide more information for guiding personalized therapy [81]. However, these combination treatments always produced serious adverse effects, especially in the case of bevacizumab and erlotinib, which caused the most adverse events of grade 3 or higher.

### 3.3. Downstream Pathway Activation and Targeting Combination Treatments

The amplification and mutation of oncogenic receptors generally leads to the activation of downstream signaling pathways that regulate cell proliferation, cell cycle progression, and cell survival. Thus, directly modulating the involved downstream factors can affect the development of acquired resistance. The involved downstream signaling pathways mainly include the RAS/RAF/MEK/ERK, PI3K/AKT/mTOR, and STAT3 pathways.

#### 3.3.1. RAS/RAF/MEK/ERK

The RAS oncogenic genes include KRAS, NRAS, and HRAS. The KRAS mutation was reported to be associated with acquired resistance, resulting in a low sensitivity to first-generation TKIs [82]. The specific role of KRAS in acquired resistance is still under investigation, and no effective KRAS inhibitor has yet been produced. BRAF, a downstream kinase of RAS, also participates in acquired resistance. About 2–4% of lung adenocarcinomas patients harbor the BRAF mutation, and the observed mutations are always V600E and G469A. These mutations always trigger permanent activation of MAPK pathways and promote tumor growth [83]. 

Although rare, RTK and BRAF fusion has been detected in EGRF-mutated NSCLC patients. The rearrangement of BRAF leads to the lack of an N-terminal inhibitory domain in BRAF, resulting in the constitutive dimerization of RAF proteins and the activation of downstream MAPK signaling independently of RAS activation [84]. Novel fusions, such as PJA2/BRAF and SALL2/BRAF fusions, that were recently discovered were also demonstrated to mediate resistance to EGFR-TKIs, providing a potential mechanism of TKI resistance [85].

#### 3.3.2. RAS/RAF/MEK/ERK-Targeted Strategies

Since KRAS mutation is negatively correlated with the efficacy of TKI treatment, there are few effective targeted drugs for these patients. Thus, chemotherapy is still the primary therapy for KRAS mutations. The development of BRAF mutation-targeted therapy was slow until 2017, when the combination of dabrafenib (BRAF V600E inhibitor) plus trametinib (MEK inhibitor) was approved by the FDA for the treatment of patients with the BRAF V600E mutation. The ORRs of this treatment in patients with previously treated and untreated BRAF-mutant NSCLC cancer were 63% and 61%, respectively, which demonstrates that it is an effective therapy with meaningful activity [86]. Other combination strategies involving MEK inhibitors have also been explored. The antitumor effect of AZD6244 (a MEK inhibitor) plus gefitinib was tested in NSCLC cell lines that harbor NRAS/KRAS mutations, showing a greater growth inhibition than single-drug treatment [87,88]. Other MEK inhibitors, such as CI1040 and CZ0775, show the same trend and, hence, a better combination regime needs to be developed [89].

#### 3.3.3. PI3K/AKT/mTOR

PTEN is a kinase that can inhibit the activation of PI3K/AKT by dephosphorylating PIP-3 to PIP-2. The loss of PTEN is the mechanism for TKI resistance via reactivating AKT. A high expression of PTEN was proven to restore the efficacy of TKIs in TKI-resistant cell lines [90]. Another factor that can lead to sustained PI3K/AKT pathway activation is the *PIK3CA* mutation. The *PIK3CA* gene is responsible for coding the catalytic subunit of PI3K, and its mutation was first demonstrated by Sequist to affect TKI-resistance progression in NSCLC patients [91]. *PIK3CA* mutations are always observed as coexisting with EGFR mutations.

#### 3.3.4. PI3K/AKT/mTOR-Targeted Combination Treatment

Most of the combination treatments involving PI3K/AKT/mTOR inhibitors have only been studied at the preclinical stage. For example, the combination of the PI3K/AKT inhibitors BEZ235 and LY294002 with erlotinib and gefitinib, respectively, can restore sensitivity to TKIs in TKI-resistant cell lines [92,93]. KU-0063794, a potent and specific mTOR inhibitor, can enhance the antitumor activity of erlotinib in TKI-sensitive PC9 cells and overcome erlotinib resistance in TKI-resistant H1650 cells [94].

Apart from that, a feedback loop exists between the MEK/ERK and PI3K/AKT pathways in EGFR-TKI resistant cell lines; thus, single-agent treatment may not be effective. To overcome this problem, the combined inhibition of MEK plus PI3K was investigated in EGFR-TKI-resistant cell lines. The results showed that the combination of trametinib plus taselisib successfully inhibited the compensatory activation of intracellular signals and cells growth [95].

#### 3.3.5. STAT3

STAT3 is member of the signal transducer and activator of transcription family of transcription factors. It can be phosphorylated by JAK and then dimerized and moved into the nucleus to regulate the cell fate. STAT3 can be activated by cytokine-like growth hormones and IL-6 family cytokines, and some loops may occur amongst them. The IL-6/STAT3 pathway was reported to be activated to maintain cell survival after EFGR-TKI treatment. In addition, an autocrine loop of IL-6/IGF-1R/STAT3 was recently reported to play a role in EMT-mediated TKI resistance [96]. Moreover, the coordinated activation of YAP1 signaling by IL-6 may mediate TKI resistance [97]. A better understanding of the signaling network is necessary and would assist in the improved design of targeted therapy for patients experiencing drug resistance.

#### 3.3.6. STAT3-Targeted Combination Treatment

Preclinical studies reported that the combination of JAK2 inhibitors (AZD1480) can restore cell sensitivity to TKIs via uncoupling EGFR from its negative regulatory proteins, SOCS4/5 proteins, in TKI-resistant EGFR cell lines [98]. Pacritinib, a JAK2/FLT3 inhibitor, can exhibit synergistic antitumor effects when combined with erlotinib in TKI-resistant cell lines [99]. A phase Ib study investigated the efficacy of combination of afatinib plus ruxolitinib, a JAK1/2 selective inhibitor, in NSCLC patients with progression following treatment with EGFR-TKIs. Although frequent adverse events including diarrhea, acneiform rash, anemia, and paronychia were observed, the combination strategy was tolerated by patients. However, the clinical activity was modest in NSCLC patients with acquired resistance to EGFR-TKIs, with an ORR and mPFS of 23.3% and 4.9 months, respectively [100].

### 3.4. Histological/Phenotypic Transformation

#### 3.4.1. Small Cell Transformation

About 5–10% patients present histologic transformation from NSCLC to small-cell lung cancer (SCLC) during or after treatment with EGFR-TKIs [101]. Although the mechanism of histological transformation still remains largely unknown, possible therapy involves the loss of the retinoblastoma gene (RB) that may participate in this process. RB deficiency is ubiquitous in SCLC and SCLC transformation and appears predisposed in NSCLC via inactivating RB and p53 [102]. Thus, RB1 and Tp53 can serve as predictive biomarkers for SCLC transformation. However, small-cell transformation is still a tricky problem that needs to be solved. Tumor re-biopsy and the analysis of clinical data are required to study the mechanism.

#### 3.4.2. Epithelial to Mesenchymal Transition

Epithelial–mesenchymal transition (EMT) has some profound effects on both morphology and physiology. During this process, cancer cells lose epithelial characteristics through the loss of E-cadherin, leading to increased vimentin expression and transformation into the mesenchymal phenotype. Emerging evidence has demonstrated an association between EMT and TKI drug resistance. The gefitinib-resistant HCC827 and PC9 cell lines undergo EMT, transitioning to the mesenchymal phenotype [103]. A recent study also revealed that osimertinib-resistant H1975 cells exhibit EMT characteristics in the absence of other EGFR mutations [104]. EMT is an orchestrated process involving various regulators, such as EMT-inducing transcription factors (EMT-TFs), noncoding RNAs, and various extracellular signal factors. EMT-TFs play important roles during all stages of the EMT process. The most well-known EMT-TFs are members of the SNAIL, ZEB, and TWIST families. Many studies have shown that the overexpression of SLUG and SNAIL can induce drug resistance [105]. The reversion of EMT can contribute to recovery of gefitinib sensitivity.

#### 3.4.3. miRNAs and EMT

**Figure 2:**
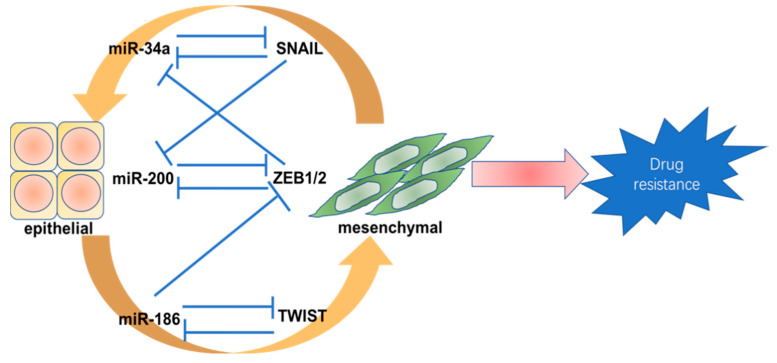
The relationship between miRNAs and epithelial–mesenchymal transition-inducing transcription factors (EMT-TFs).

Apart from EMT-TFs, long noncoding RNAs (lncRNAs) and miRNA play an important role in regulating EMT and TKI resistance. After long-term gefitinib treatment, the expression levels of miR-155 and miR-200c declined significantly [106]. Some miRNAs are associated with EMT-TFs and form feedback loops to coregulate EMT and drug resistance. The most common miRNAs that were shown to act as inhibitors of EMT are the miR-200 and miR-34 families [107]. Various loops exist among EMT-TFs and miRNAs, as can be seen in Figure 2. miR-200, for example, can target ZEB1 and inhibit its synthesis, then repress the transcription of miR-200 by binding to its promoter, resulting in a double-negative feedback loop. Most miR-200 members only target ZEB2, whereas miR-200a and miR-200b can target both ZEB1 and ZEB2 to form a double-negative feedback loop [108]. A similar double-negative feedback loop can be formed by miR-34a and SNAIL. Other miRNAs, like miR-1, miR-29b, and miR-203, can also regulate EMT-TFs and then negatively regulate EMT. Although most miRNAs are found to inhibit EMT, some miRNAs exhibit characteristics of promoting EMT, including miR-21 and miR-155, amongst others [109,110]. Some miRNAs can promote TKI resistance via activating the PI3K/AKT/mTOR signaling pathways. miR-21 and miR-23a can target PTEN and then activate AKT, resulting in resistance to TKIs in NSCLC cells [111,112]. However, EMT is a highly dynamic and continuous process and cannot be simply be defined as a binary transition. Determining how to regulate the equilibrium of EMT might provide new possibilities for drug resistance therapy.

#### 3.4.4. EMT/miRNA-Targeted Combination Treatment

Since both EMT and miRNAs play roles in acquired resistance and various loops exist among EMT, EMT-TFs, and miRNAs, combination treatment that targets those factors could be considered. However, most of these combination strategies are still under preclinical study. Zhuo et al. showed that cisplatin-induced apoptosis can be enhanced in TWIST siRNA-treated cells [113]. Gou et al. reported that the combination of crizotinib plus the downregulation of TWIST1 using specific siRNA can remarkably improve the sensitivity of cells to crizotinib [114]. Apart from that, miR-200/ZEB was demonstrated to regulate sensitivity to nintedanib, a multi-target angiokinase inhibitor, in NSCLC cells [115]. The results suggested that nintedanib reverses TGF-induced EMT and resistance to gefitinib via regulating the miR-200/ZEB axis. The specific targeting of the miR-200/ZEB axis by this therapy should be further confirmed in future studies. A recent study reported evidence of the involvement of miR-483-3p and EMT in drug resistance using both an in vivo and in vitro EGFR-mutant NSCLC model with TKI resistance. The inhibition of miR-483-3p can efficiently restore the sensitivity of gefitinib-resistant cells to gefitinib via reversing EMT and inhibiting proliferation, migration, and invasion [116]. This suggested that miR-483-3p is a promising potential target for combination therapy to overcome acquired resistance. Moreover, miRNAs can improve the sensitivity to EGFR-TKIs not only via regulating EMT but also through other signaling pathways. For example, miR-641 was found to activate ERK signaling by directly targeting neurofibromatosis. Hence, the combination of miR-641 inhibition plus erlotinib remarkably inhibited erlotinib-resistant NSCLC cell growth in mice [117]. Taken together, this demonstrates the importance in understanding the mechanisms of acquired resistance and identifying the loops among those factors, which can provide more potential targets for specific individual therapy.

## 4. Other Combination Treatment Strategies for Acquired Resistance

### 4.1. Immunotherapy

With the development of immunotherapy, nivolumab and pembrolizumab emerged as standard treatments. A series of preclinical studies suggested that an association exists between the EGFR mutation and PD-L1 upregulation in NSCLC. PD-L1 inhibition was demonstrated to improve survival in an EGFR-driven cancer mouse model via the enhancement of effector T cell function, which provides the possibility of combination therapy of PD-1/PD-L1 inhibitor plus EGFR-TKIs as a treatment for NSCLC patients [118]. Several clinical studies of these combination treatments have been conducted. The combination of erlotinib plus atezolizumab in EGFR-TKI-naive patients showed an ORR and PFS of 75% and 11.2 months, respectively [119]. In a phase Ib study, patients with advanced TKI therapy were treated with osimertinib plus durvalumab, and the ORRs were 67% and 21% for T790M positive and T790M negative, respectively [120]. Although some results showed the potential efficacy of those strategies, overlapping toxicities should be a concern when considering use in patients. For example, the frequency of interstitial pneumonitis in the combination treatment of EGFR-TKI plus nivolumab (25.7%) is higher than single EGFR-TKI treatment (4.59%) [121]. Some patients have to discontinue the combination treatment due to experiencing serious adverse events. Thus, a careful evaluation will be required to further develop combination therapy.

### 4.2. Antidiabetic Drugs

Metformin can improve the sensitivity to EGFR-TKIs in patients with diabetes and NSCLC. Thus, whether it can enhance the effect of TKIs was studied in both preclinical and clinical research. Studies showed that metformin can effectively increase the sensitivity to erlotinib and gefitinib in TKI-resistant cell lines via reserving EMT and decreasing IL-6 signaling activation [122]. In a randomized, double-blind phase II trial, EGFR-mutant NSCLC patients without diabetes were randomly assigned in a 1:1 ratio to receive gefitinib plus either metformin or placebo. Non-significant worse outcomes were observed in the metformin group, although the values for mPFS (10.3 months vs. 11.4 months) and mOS (22.0 months vs. 27.5 months) were lower in the metformin group [123]. In another randomized phase II clinical trial, patients with confirmed stage IIIB–IV lung adenocarcinoma were randomly allocated to receive EGFR-TKI (erlotinib, afatinib, and gefitinib) plus metformin or TKI alone. The results showed that both mPFS (9.8–163 months vs. 7.5–12.2 months) and mOS (20.5–42.8 months vs. 11.4–23.7 months) were significantly longer in the patients receiving combination treatment than single-agent treatment [124]. Although these two studies showed some differences, this combination strategy seems feasible for patients, but the efficacy needs to be confirmed.

### 4.3. Combination Chemotherapy

For patients who suffer from progressive disease after TKI treatment, chemotherapy is still their only option. The combination of EGFR-TKIs plus chemotherapy may provide an alternative approach to delay or overcome acquired resistance. To date, various chemotherapy combination treatments were studied in clinical settings; these strategies mainly included first-generation EFGR-TKIs plus a pemetrexed/docetaxel/platinum-based regimen (Table 3). The efficacy of the concurrent combination of platinum-based chemotherapy plus gefitinib was tested in treatment-naïve NSCLC patients with sensitive mutations in phase II NEJ005/TCOG0902 clinical trials [125]. In 2018, the results demonstrated that the concurrent combination of carboplatin-pemetrexed plus gefitinib showed a significantly longer OS (41.9 vs. 30.7 months) and better PFS (17.5 vs. 15.3 months) than a sequential or alternating strategy where patients were treated with alternating gefitinib for eight weeks and two cycles of pemetrexed [126]. In a randomized phase III study (NEJ009), the difference in the efficacy between the combination of carboplatin-pemetrexed plus gefitinib and gefitinib alone was assessed. A longer PFS (20.9 vs. 11.2 months), longer OS (52.2 vs. 38.8 months), and better RR (84% vs. 67.4%) was observed for the combination treatment [127,128]. However, for patients who progressed to first-line EGFR-TKI therapy, there was no difference in mPFS between the combination treatment and chemotherapy alone in patients who harbored the T790M mutation. T790M-negative patients could benefit from combination treatment, with an mPFS of 6.7 vs. 5.4 months [129].

For the combination of third-generation TKI plus chemotherapy, little clinical data are available and only a few cases have been reported. For example, Yoshida et al. successfully treated a patient with the combination of osimertinib plus chemotherapy with carboplatin, pemetrexed, and bevacizumab. At ASCO 2018, Okada et al. reported a good safety profile for osimertinib plus carboplatin-pemetrexed combination treatment in 24 T790M-positive NSCLC patients who progressed to treatment with EGFR-TKI [130]. Clinical trials regarding osimertinib plus chemotherapy, such as FLUARA2 (NCT04035486) and TAKUMI (LOGIK1604/NEJ032A), are still ongoing.

### 4.4. Other Combination Treatment

The use of other drugs or inhibitors in combination strategies has also been studied. For example, YM155 is a small-molecule survivin inhibitor that can induce cell apoptosis and autophagy. Research has demonstrated that YM155 can effectively enhance the sensitivity of erlotinib in EGFR-resistant H1650 and A549 cell lines. The synergistic effect of the combination of YM155 plus erlotinib was observed in a mouse model, resulting in the inhibition of EGFR-mutant tumors [131]. Hsp90 is a chaperone protein critical for cellular survival. The combination of its inhibitors (onalespib, ganetespib, and 17-AAG) plus TKIs exhibited remarkable antitumor effects in preclinical studies [132,133,134]. For instance, the growth of tumor regressed was greater for the combination of erlotinib plus onalespib than either monotherapy in EGFR-driven xenograft tumors [132]. HDAC inhibitors exert potential antitumor effects via the induction of cell cycle arrest, differentiation, or apoptosis, as demonstrated both in vitro and in vivo. The combination of vorinostat and newer-generation TKIs (WZ4002/BIBW2992) was reported to induce a synergistic anticancer effect both in vitro and in vivo via the autophagy-mediated activation of apoptosis in H1975 cells [135]. In phase I/II clinical trials, these strategies did not show appreciable antitumor activities in EGFR-mutated NSCLC patients with acquired resistance [136].

## 5. Conclusions

The standard first-line treatment for EGFR-mutated NSCLC patients is the use of EGFR-TKIs. However, the resistance acquired after EGFR-TKI treatment is a complex problem that needs to be solved. The different mechanisms of acquired resistance found in various patients must all be identified such that the optimal treatment can be chosen based on these specific mechanisms. To prevent acquired resistance, sequential therapy can be considered according to the specific EGFR mutation. The continuation of EGFR-TKI therapy and combination therapy are feasible for patients with disease progression. Repeated tumor biopsy and liquid biopsy are two main methods for detecting the EGFR T790M mutation. Combination approaches have been investigated in the treatment of patients with acquired resistance. To determine the optimal combination treatment, the genetic signature in patients should be confirmed. Although some combinations strategies produce some better clinical outcomes, the majority are still under development. Some combination treatments that showed encouraging results in the preclinical stage failed to translate into favorable clinical outcomes. Hence, more studies should be conducted in the future to enrich our understanding of the complex oncogenic signaling pathways involved in acquired resistance, leading to improved combination treatments in these cases.

## Figures and Tables

**Table 1:** Epidermal growth factor receptor-tyrosine kinase inhibitor (EGFR-TKIs) approved by the FDA.

Generation	EGFR-TKI	Inhibition	Molecular Target	Adverse Events
1st	Gefitinib	Reversible	EGFR del19, L858R	Skin rash/acne, abnormal liver function test, anorexia, stomatitis diarrhea, paronychia
	Erlotinib	Competitive	EGFR del19, L858R	Rash, diarrhea, edema, cough, conjunctivitis
2nd	Afatinib	Irreversible; covalent	EGFR, HER2, HER4	Diarrhea, skin rash, paronychia, stomatitis
3rd	Osimertinib	Irreversible; covalent	EGFR mutations and T790M	Skin rash, diarrhea, ILD, QTc prolongation, ocular disorder cardiomyopathy

Abbreviations: QTc, QT interval corrected for heart rate; ILD, interstitial lung disease.

**Table 2:** Drugs in use or in development for acquired resistance mechanisms.

Mechanism of Resistance	Potential Targets for Combination Strategies	Targetable Drug
HER2 amplification/mutation	HER2	Afatinib, Trastuzumab, Dacomitinib Trastuzumab emtansine (T-DM1)
HGF/MET axis amplification/mutation	MET	Selective MET inhibitor: SHR-A1403, Tivantinib (ARQ197), Capmatinib (INC280), Savolitinib (AZD6094). Tepotinib Multikinase inhibitors: Crizotinib, Cabozantinib, Glesatinib,
	HGF	Rilotumumab, Ficlatuzumab
AXL amplification	AXL	YD, E8, D9 Cabozantinib, Sitravatinib (MGCD516), Bemcentinib (R428)
FGFR1 amplification		SU5402, PD166866
RAS/RAF/MEK/ERK	BRAF	Dabrafenib (GSK2118436), Vemurafenib (PLX4032)
	MEK	Trametinib (GSK1120212), Selumetinib (AZD6244), CI1040
PI3K/AKT/mTOR	PI3K	Pilaralisib (XL147), Dactolisib (BEZ235), Pictilisib (GDC-0941), Buparlisib (BKM120), LY294002
	AKT mTOR	MK-2206 Everolimus (RAD001), Temsirolimus (CCI-779), Ridaforolimus (MK-8669), KU-0063794
JAK/STAT3	JAK	AZD1480, Pacritinib, Ruxolitinib

**Table 3:** Combination treatments of chemotherapy plus EGFR-TKIs.

Trial Phase	Treatment Regimens	No. of Patients	ORR	mPFS (Months)	mOS (Months)
II	Gefitinib + carboplatin + pemetrexed (concurrent vs. sequentially alternating)	80	90.2% vs. 82.1%	17.5 vs. 15.3	41.9 vs. 30.7
III	Gefitinib + carboplatin + pemetrexed vs. Gefitinib	344	84.0% vs. 67.4%	20.9 vs. 11.2	52.2 vs. 38.8
III	Gefitinib + carboplatin/pemetrexed vs. Gefitinib	350	75.3% vs. 62.5%	16.0 vs. 8.0	- vs. 17.0
II	Gefitinib + pemetrexed vs. Gefitinib + placebo	90	80.0% vs. 73.0%	18.0 vs. 14.0	34.0 vs. 32.0
II	Erlotinib + docetaxel/pemetrexed vs. docetaxel/pemetrexed	46	17.0% vs. 13.0%	4.4 vs. 5.5	14.2 vs. 16.4
II	Gefitinib + pemetrexed vs. gefitinib	191	-	16.2 vs. 11.1	43.4 vs. 36.8

## Abbreviations

NSCLC	non-small-cell lung cancer
ErbB	erythroblastic oncogene B
EGFR	epidermal growth factor receptor
PTEN	phosphatase and tensin homolog
PI3K	phosphoinositide 3-kinases
MEK	mitogen-activated protein kinase kinase
ERK	extracellular signal-regulated kinases
TKIs	tyrosine kinase inhibitor
RTKs	receptor tyrosine kinases
MET	mesenchymal–epithelial transition factor
HGFs	hepatocyte growth receptors
IGFRs	insulin-like growth factors receptors
FGFRs	fibroblast growth factor receptors
VEGFRs	vascular endothelial growth factor receptors
MET	tyrosine-protein kinase Met
PIK3CA	phosphatidylinositol-4,5-bisphosphate 3-kinase, catalytic subunit alpha
HGF	hepatocyte growth factor
FGFR	fibroblast growth factor receptors
JAK	Janus kinase
MAPK	mitogen-activated protein kinase
SPRY4	Protein sprouty homolog 4
VEGF	Vascular endothelial growth factor
PJA2	E3 ubiquitin-protein ligase Praja2
SNAIL2	Sal-like protein 2
RR	response rate
ORR	objective response rate
PFS	Progression Free Survival
mPFS	median PFS
OS	overall survival
mOS	median OS
DCR	Disease Control Rate
FDA	Food and Drug Administration
ILD	Interstitial lung disease
QTc	QT interval corrected for heart rate
CNS	central nervous system
EGFR-TFs	EMT-inducing transcription factors
